# Nightingale: web components for protein feature visualization

**DOI:** 10.1093/bioadv/vbad064

**Published:** 2023-05-24

**Authors:** Gustavo A Salazar, Aurélien Luciani, Xavier Watkins, Swaathi Kandasaamy, Daniel L Rice, Matthias Blum, Alex Bateman, Maria Martin

**Affiliations:** Macromolecules, Structure, Chemistry and Bioimaging Section, European Bioinformatics Institute, European Molecular Biology Laboratory (EMBL-EBI), Wellcome Genome Campus, Hinxton, CB10 1SD, UK; Macromolecules, Structure, Chemistry and Bioimaging Section, European Bioinformatics Institute, European Molecular Biology Laboratory (EMBL-EBI), Wellcome Genome Campus, Hinxton, CB10 1SD, UK; Macromolecules, Structure, Chemistry and Bioimaging Section, European Bioinformatics Institute, European Molecular Biology Laboratory (EMBL-EBI), Wellcome Genome Campus, Hinxton, CB10 1SD, UK; Macromolecules, Structure, Chemistry and Bioimaging Section, European Bioinformatics Institute, European Molecular Biology Laboratory (EMBL-EBI), Wellcome Genome Campus, Hinxton, CB10 1SD, UK; Macromolecules, Structure, Chemistry and Bioimaging Section, European Bioinformatics Institute, European Molecular Biology Laboratory (EMBL-EBI), Wellcome Genome Campus, Hinxton, CB10 1SD, UK; Macromolecules, Structure, Chemistry and Bioimaging Section, European Bioinformatics Institute, European Molecular Biology Laboratory (EMBL-EBI), Wellcome Genome Campus, Hinxton, CB10 1SD, UK; Macromolecules, Structure, Chemistry and Bioimaging Section, European Bioinformatics Institute, European Molecular Biology Laboratory (EMBL-EBI), Wellcome Genome Campus, Hinxton, CB10 1SD, UK; Macromolecules, Structure, Chemistry and Bioimaging Section, European Bioinformatics Institute, European Molecular Biology Laboratory (EMBL-EBI), Wellcome Genome Campus, Hinxton, CB10 1SD, UK

## Abstract

**Motivation:**

The visualization of biological data is a fundamental technique that enables researchers to understand and explain biology. Some of these visualizations have become iconic, for instance: tree views for taxonomy, cartoon rendering of 3D protein structures or tracks to represent features in a gene or protein, for instance in a genome browser. Nightingale provides visualizations in the context of proteins and protein features.

**Results:**

Nightingale is a library of re-usable data visualization web components that are currently used by UniProt and InterPro, among other projects. The components can be used to display protein sequence features, variants, interaction data, 3D structure, etc. These components are flexible, allowing users to easily view multiple data sources within the same context, as well as compose these components to create a customized view.

**Availability and implementation:**

Nightingale examples and documentation are freely available at https://ebi-webcomponents.github.io/nightingale/. It is distributed under the MIT license, and its source code can be found at https://github.com/ebi-webcomponents/nightingale.

## 1 Introduction

The advances in the understanding of life sciences have often been accompanied by engaging visualizations: from Darwin, with his tree of life, to AlphaFold structural predictions with its striking coloring of confidence. Visualizations have been used for teaching the fundamentals of biology (e.g. in school textbooks), and they have also been used to explain the most cutting-edge developments, as you can appreciate by browsing any recent issue of a biology journal.

Visualization is an active field of research and techniques have improved over time, with new strategies on how to represent data in the best way possible for each different case. A driving factor in this evolution has been technological. Thanks to this, visualizations are now not limited to paper and can be found in all types of displays, from the small screens of a cell phone to fully immersive virtual reality. Besides the medium, the biggest improvement is interactivity. These days visualizations can allow the audience to engage with the data directly and control some elements: filtering data, focusing on particular areas, coloring or marking pieces of the visualization, etc. Another factor to consider is the ubiquity of the web, which makes it the perfect environment to host visualizations: the audience is now global, and there are technical standards for how to render graphics, and manipulate their parts; to create interactive, performant and beautiful visualizations.

The purpose of Nightingale is to take advantage of these standards, to create a library of biological web components that can be reused by multiple teams and projects, streamlining some visual aspects and allowing the users to focus on their data and research questions.

Unsurprisingly, Nightingale is not the only project working in this scope. There is an increasing number of web visualizations available for bioinformatics projects looking to explore, understand and explain complex biological systems (Wang *et al.*, 2015).

However, Nightingale is not intended as a solution for all biological visualization needs, but rather its main biological focus is on the visualization of proteins, their features, domains and other regions of interest.

ProtVista (Watkins *et al.*, 2017) is Nightingale’s predecessor. It was developed and maintained by UniProt (The UniProt Consortium, 2022) between 2013 and 2018. ProtVista was a visualization tool for the graphical representation of protein sequence features in the UniProt Knowledgebase, and it was shared with the community as a BioJS component. BIoJS is a project that takes bioinformatics visualization components published in npm and presents them in a searchable interface, together with their documentation and examples (Corpas *et al.*, 2014).

In 2018, during the development of a new version of the InterPro website (Paysan-Lafosse *et al.*, 2023), it became clear that InterPro required a tool similar to ProtVista, however, it was also evident that it was going to require a lot of work to adapt it to the particular needs of the InterPro project. We then started a collaboration between the two teams, to modularize ProtVista and use web standards to create a set of reusable components, able to answer the needs of both projects, but purposely generic enough for other projects to adopt or extend. The outcome of this collaboration became what is currently referred to as Nightingale components.

Both UniProt and InterPro use Nightingale components in their websites, and other projects have adopted and extended them. In addition, the scope of the components has grown, and now there are Nightingale components to visualize heatmaps, taxonomy, multiple sequence alignments and more.

The main difference between ProtVista and Nightingale is the modularity of the latter. While ProtVista was a single visualization unit, the components in Nightingale can be added or removed in order to customize the protein viewer for the particular needs of the use case.

## 2 Methods

Nightingale components are modular, so the user can display a single track, or combine multiple components to create their own personalized visualization. All components are published in npm (https://www.npmjs.com/search?q=keywords%3Anightingale%20webcomponents).

Nightingale follows web standards, and all its components are developed as custom elements following the HTML specification authored by W3C and WHATWG (https://html.spec.whatwg.org/).

Nightingale is open source, and our code resides in GitHub at https://github.com/ebi-webcomponents/nightingale, and although the main developers are from UniProt and InterPro, we have received contributions from software developers from several institutions.

Nightingale is a monorepo: a single repository containing all the components as packages. In this way the common tasks, such as bundling, deployment and testing can be centralized, while the details of each component are isolated per package. It uses Lerna (https://lerna.js.org/) to coordinate this approach.

Nightingale is well documented and provides code examples, the reference for which can be found in the README.md file that is part of each package. This makes it available from GitHub, but also it is used to generate the documentation area in Nightingale’s website.

Nightingale version 4.0.0 was released in March 2023. It is a complete re-factor of the common functionality. The new version has been rewritten in TypeScript (https://www.typescriptlang.org/), which helps with the early detection of bugs, and improves the developer experience when used in combination with a good integrated development environment (IDE). In this version, we used Lit (https://lit.dev/) as a minimalistic framework for the components. This reduces the need for some boilerplate code associated with web components and offers some tools to better organize the code. In particular, we use Lit’s implementation of mixins to create small functionalities that each component can opt into.

Following modern practices of web development, Nightingale components are now deployed as es-modules, a feature that is currently supported on all major browsers, and that allows the creation of web applications that combine multiple components without the need for a bundler that pre-processes the files.

The new packages are published in npm using the namespace ‘@nightingale-elements’: https://www.npmjs.com/org/nightingale-elements which makes it easier to identify them as an official nightingale element.

## 3 Results

### 3.1 Published components

**Fig. 1.**
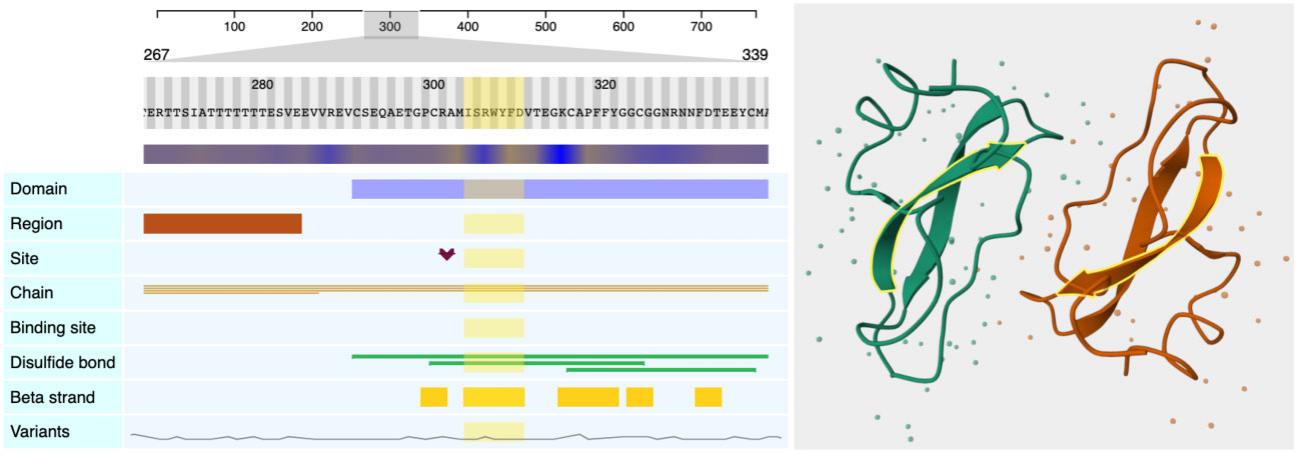
Snapshot of a composition of several nightingale components to display available information in UniProt for the Amyloid-beta precursor protein (UniProtKB: P05067, PDB: 1AAP)

Figure 1, on the left side, shows several Nightingale track components visualizing UniProt data for the Amyloid-beta precursor protein (UniProt accession: P05067), and on its right, one of the structures associated with this protein (PDB accession: 1AAP) using the nightingale-structure component.

The current release of Nightingale includes the following track components, all of which have common attributes to select a region on a protein of a given length:

sequence^†^: Displays a sequence of amino acids.

colored-sequence: Paints a color depending on each residue in the sequence.

track^†^: Renders protein features as simple shapes given their coordinates.

interpro-track: Extends track in order to represent the domain composition intrinsic to InterPro entries.

linegraph-track: Displays line graphs whose values correspond to positions in a protein sequence.

links: A node-link representation to visualize points of interaction between bases in a sequence.

msa: Multiple Sequence Alignment viewer, using the same coordinate system that other tracks.

structure: A wrapper over Mol* (Sehnal *et al.*, 2021) to display protein structures. Although, it is not displayed as a track, it shares the same logic, in order to support interactivity with other tracks.

Nightingale also includes a set of utility components, supporting different functionalities in the protein feature viewer:

manager^†^: Container for all the tracks, and facilitates communication between them.

navigation^†^: Toolbar to zoom and navigate along the sequence.

saver: Takes snapshots of the current viewer as a PNG image.

overlay: Creates a layer over a given HTML element. Used to inform the user they need to press CTRL to zoom.

There are two components that don’t fit in the context of the protein viewer: sunburst to display taxonomy data, and heatmap currently used to display confidence levels of structure models.

In addition, we created a meta-component called protvista-uniprot. This is a single package that covers all the features integrated into the original ProtVista component, but in a more configurable way. It combines the essential components^†^ and some of the other utilities. This has also served as a template for projects that want to include a customized version of ProtVista such as Pharos (Sheils *et al.*, 2021).

### 3.2 Components adoption

Multiple projects are currently using Nightingale components and Table 1 lists some of these.

**Table 1.** Projects using Nightingale components

Project	Example URL	Components Used
InterPro	https://www.ebi.ac.uk/interpro/protein/UniProt/P05067/	All components, except structure
UniProt	https://www.uniprot.org/uniprotkb/P05067/feature-viewer	All components, except interpro-track and links
PDBe	https://www.ebi.ac.uk/pdbe/entry/pdb/1aap/protein/1	Extends protvista-uniprot using Essential components^†^ and develop its own components
Enzyme Portal	https://www.ebi.ac.uk/enzymeportal/search/P31153/enzyme	Uses protvista-uniprot
Pharos	https://pharos.ncats.nih.gov/targets/ULK4#sequence	Uses protvista-uniprot and develop its own components
GlyGen	https://glygen.org/protVista/P46782	Essential components^†^

† Essential components have been marked as such in section 3.1.

Nightingale components have proven to be beneficial not only for the projects leading its development, but also for the community that shares similar visualization needs. It is important to highlight that members of this community have contributed back to the project with bug fixes, adding features, reporting errors, etc. Thanks to these contributions we have been able to improve the components over time, making them more generic and robust.
